# Self-compassion mediates the relationship between dispositional mindfulness and athlete burnout among adolescent squash players in South Africa

**DOI:** 10.17159/2078-516X/2021/v33i1a11877

**Published:** 2021-10-28

**Authors:** SP Walker

**Affiliations:** Department of Psychology, University of the Free State, Bloemfontein, South Africa

**Keywords:** reduced accomplishment, sport devaluation, youth athletes, mediation

## Abstract

**Background:**

Dispositional mindfulness has been found to positively impact athlete burnout. Furthermore, self-compassion has been identified as a potential mechanism of action through which mindfulness is related to lower rates of athlete burnout. However, this interaction has yet to be investigated among adolescents.

**Objectives:**

To determine whether self-compassion mediates the relationship between dispositional mindfulness and athlete burnout among adolescent squash players in South Africa.

**Methods:**

Competitive adolescent squash players (n=158) from two provinces in South Africa completed measures of dispositional mindfulness, self-compassion and athlete burnout. Intercorrelations were calculated between the three variables. An ordinary least squares regression analysis was performed to test the indirect effect of self-compassion on the relationship between dispositional mindfulness and the three components of athlete burnout.

**Results:**

Both dispositional mindfulness and self-compassion were negatively related to athlete burnout, while displaying positive correlations with each other. Self-compassion was found to partially mediate the relationship between dispositional mindfulness and a sense of reduced accomplishment (*b* = −0.075; 95% CI [−0.037; −0.012]), as well as the association between dispositional mindfulness and sport devaluation (*b* = −0.056; 95% CI [−0.099; −0.022]). The relationship between dispositional mindfulness and exhaustion was, however, not mediated by self-compassion (*b* = −0.002; 95% CI [−0.052; 0.049]).

**Conclusion:**

The effect of dispositional mindfulness on certain components of athlete burnout is partially mediated by self-compassion among adolescent athletes. Based on the current findings, interventions aimed at increasing mindfulness among adolescent athletes appear to be a potential avenue by which to reduce certain aspects of burnout, partially through increasing self-compassion.

Athlete burnout (ABO) is a multifaceted phenomenon characterised by physical and/or mental exhaustion, a reduced sense of accomplishment from participating in sport, and a tendency to devalue the role of sport in the individual’s life.^[1]^ The demands placed upon adolescent athletes with respect to education, peer relationships, identity development, training and competition, as well as satisfying parental and coaching expectations puts this population at risk for ABO.^[2]^ ABO is associated with short-term performance and mental health costs, as well as a reduction in lifetime physical activity. ^[3]^ Consequently, psychological processes implicated in the development and modulation of ABO among adolescent athletes warrant greater research attention.

Evidence of an inverse relationship between dispositional mindfulness (DM) and ABO continues to mount across athlete populations.^[4]^ DM, a relatively stable awareness of the present moment, is characterised by acceptance and non-judgement towards internal experience and external stimuli, and is increasingly being shown to positively impact athletic performance and emotional well-being among athletes.^[5]^ DM is viewed as being primarily trait-like and is often differentiated from cultivated mindfulness, which is the state more commonly associated with mindfulness training and meditation practice.^[5]^ Despite the connection between DM and ABO, little is known about the psychological processes by which DM might impact ABO. It has been hypothesised that DM may enhance athlete performance and well-being through increased task-focussed attention, greater acceptance of external influences and internal experiences, more effective emotional regulation, a reduced tendency to ruminate and the facilitation of psychological flexibility.^[6]^ However, this is based almost exclusively on data from clinical and non-athlete populations.

Self-compassion (SC) has been proposed as a possible mechanism that might augment or modulate the impact of DM on psychological well-being in the general population.^[7]^ SC is broadly conceptualised as an attitude towards the self that is characterised by the absence of self-judgement, self-kindness, a sense of connection with others based on common human experiences, and a mindful orientation towards events and experiences.^[8]^ Within the sporting realm, SC has been associated with improved psychological well-being and an increase in perceived performance among Canadian female collegiate athletes.^[9]^ It has also been suggested that SC may augment and/or modulate the interaction between DM and ABO. A recent study found that both SC and DM were inversely related to ABO among Japanese collegiate athletes.^[10]^ SC partially mediated the relationship between DM and ABO only among female participants. While DM was directly associated with SC and negatively related to ABO in the male participants, SC was not directly associated with ABO in these participants ^[10]^. In addition, female athletes reported lower levels of DM and SC compared to their male counterparts.^[10]^

Higher levels of DM appear to be associated with reduced ABO among athletes.^[4]^ A recent study reported SC as a potential mechanism that might mediate this relationship.^[10]^ However, the study was conducted among young adult collegiate athletes. Consequently, the current study aims to determine whether SC mediates the relationship between DM and ABO among adolescent athletes.

## Methods

### Participants

Ethics approval was granted by the General Human Research Ethics Committee at the University of the Free State (UFS-HSD2019/063/1007) for this study. Permission to collect data at South African Schools Squash tournaments held in two provinces was obtained from the relevant national and regional governing bodies. All 18-year-old participants, as well as the guardians of minors, provided written informed consent. The written assent of all minors was also obtained. Data were collected by means of paper-and-pencil versions of the questionnaires. All questionnaires were administered in English. Participants completed these questionnaires either after practice sessions or between matches at tournaments. Data collection was conducted in cooperation with participants and their coaches to ensure minimal disruption of training and competition.

One hundred and fifty-eight adolescent squash players (males = 108; females = 50) participated in the study. Participants ranged in age from 13 to 18 years (*M* = 15.4 years; *SD* ± 1.2). Athletes reported having played squash competitively for an average of 3.9 years (*SD* ± 2.8). At the time the data were collected, participants were practicing an average of 4.94 hours (*SD* ± 3.01) per week. The majority (56%) indicated representing their school as their highest level of participation, while 35% competed at provincial level and 10% reported being ranked among the top 10 players in their age group nationally.

### Measures

It is recommended that a multidimensional approach to measuring ABO be adopted in research.^[2]^ Consequently, the reduced sense of athletic accomplishment (RA), physical and emotional exhaustion (E) and sport devaluation (D) subscales of the Athlete Burnout Questionnaire (ABQ) were employed instead of the total score.^[1]^ Each subscale is comprised of five items. Response options ranging from “Almost never” to “Almost always” are presented along a five-point Likert-type scale. Scores for each subscale are calculated by summing responses across the five items comprising the respective subscales, with higher scores indicating greater burnout. The ABQ total score has demonstrated good reliability in a sample of adolescent South African tennis players.^[11]^ However, no local data are available with respect to the psychometric properties of the subscales.

DM was measured using the Child and Adolescent Mindfulness Measure (CAMM).^[12]^ The CAMM is a 10-item self-report inventory that yields a unitary mindfulness score, with higher scores indicating increased levels of DM. Response options ranging from “Never true” to “Always true” are presented along a five-point Likert-type scale. Adequate internal consistency has been reported for the CAMM in a sample of female adolescent hockey players in South Africa.^[13]^

The short form of the Self-Compassion Scale (SCS-SF) was employed to measure SC.^[14]^ Participants are required to respond to 12 items along a five-point Likert-type scale, with endorsement options anchored by “Almost never” and “Almost always”. After reverse scoring the negatively worded items, a total SC score is obtained by summing responses across all 12 items. Higher scores are indicative of a greater degree of SC. While no psychometric data could be found with respect to the SCS-SF in adolescent athlete samples, the original version of the SCS, from which the SCS-SF is derived, has demonstrated good internal consistency in a sample of non-athlete adolescents.^[15]^

### Statistical analysis

**Fig. 1:**
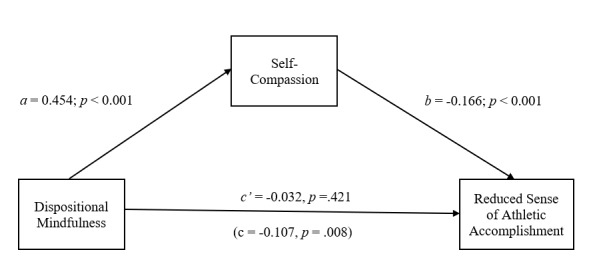
The mediating effect of self-compassion in the relationship between dispositional mindfulness and reduced sense of athletic accomplishment. All presented effects are unstandardised: a is effect of dispositional mindfulness on self-compassion; b is effect of self-compassion on reduced sense of athletic accomplishment; c′ is direct effect of dispositional mindfulness on reduced sense of athletic accomplishment; c is total effect of dispositional mindfulness on reduced sense of athletic accomplishment.

**Fig. 2:**
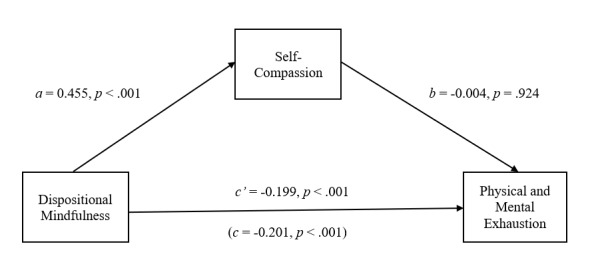
The mediating effect of self-compassion in the relationship between dispositional mindfulness and physical and mental exhaustion. All presented effects are unstandardised: a is effect of dispositional mindfulness on self-compassion; b is effect of self-compassion on physical and mental exhaustion; c′ is direct effect of dispositional mindfulness on physical and mental exhaustion; c is total effect of dispositional mindfulness on physical and mental exhaustion.

**Fig. 3:**
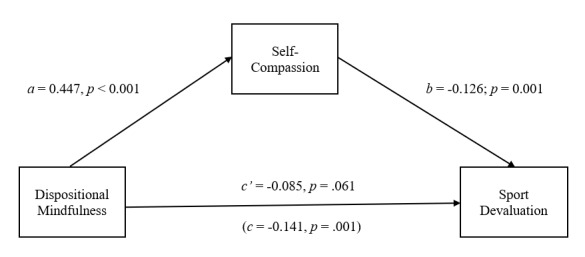
The mediating effect of self-compassion in the relationship between dispositional mindfulness and sport devaluation. All presented effects are unstandardised: a is effect of dispositional mindfulness on self-compassion; b is effect of self-compassion on sport devaluation; c′ is direct effect of dispositional mindfulness on sport devaluation; c is total effect of dispositional mindfulness on sport devaluation.

Internal consistency coefficients were calculated for all variables included in the study. Correlations between the ABQ subscales, CAMM and the SCS-SF were then calculated. Based on previous findings of a potential gender difference with respect to levels of ABO, SC and DM among athletes, a between-groups multivariate analysis of variance (MANOVA) was conducted to explore possible gender differences within the current sample.^[10]^ Finally, as it was hypothesised that SC would mediate (indirect effect) the direct effect of DM on ABO (see Figs. 1 to 3), a mediation analysis was conducted to test for the effect of SC on the relationship between DM and ABO. This was achieved through a path analytic approach employing ordinary least squares (OLS) regression analysis.^[16]^ The OLS regression analysis was performed using the PROCESS software macro for SPSS.^[16]^ A nonparametric bootstrapping method was employed to test for the statistical significance of the cross product of coefficients. Consequently, no assumptions needed to be made with respect to the distribution of ABQ, CAMM and SCS-SF scores in the sample. The regressions model was computed across 5 000 simulations utilising bias-corrected bootstrap procedures. The statistical significance of the indirect effects was determined using a 95% confidence interval.^[16]^ Analyses were conducted independently for each of the three subcomponents of ABO.

## Results

Table 1 reflects the correlations between the ABQ subscales, CAMM and SCS-SF. Mean scores, standard deviations (SDs) and internal consistency coefficients are also reported for each measure. It is apparent that internal consistency coefficients for all measures, with the exception of the ABQ-RA subscale (*α* = 0.68), meet the prescribed minimum level of acceptability for non-cognitive measures (*α* ≥ 0.70).^[17]^ Given how close this internal consistency coefficient is to satisfying the criterion, the ABQ-RA subscale was included in the subsequent analyses. All variables were statistically significantly correlated, except for the ABQ-E subscale and SCS-SF. The ABQ subscales demonstrated negative relationships with both the CAMM and SCS-SF, while the CAMM and SCS-SF were positively correlated.

A MANOVA was conducted to test for the effect of gender on DM, SC and the three components of ABO. Gender (female/male) served as the independent variable, while the ABQ-RA, ABQ-E, ABQ-D, CAMM and SCS-SF functioned as dependent variables. Preliminary assumption testing indicated no violations with respect to normality, linearity, univariate and multivariate outliers, homogeneity of variance, and multicollinearity. No significant differences were found between the female and male respondents with respect to the combined dependent variables *F* (5;148) = 1.150, *p* = 0.337, partial eta squared = 0.037.

Mediation analyses were conducted to test the direct and indirect (via SC) effects of DM on the three components of ABO. In each case, the proposed mediator (SC) was regressed on DM (path *a*), while the relevant component of ABO was regressed on SC (path *b*) and DM (path *c*′).

Fig. 1 illustrates that DM is indirectly related to ABQ-RA through SC. DM is associated with an increase in SC (*a* = 0.454, *p* < 0.001), and a decrease in ABQ-RA is related to SC (*b* = −0.166, *p* < 0.001). A bias-corrected confidence interval based on 5 000 bootstrap samples indicates that the indirect effect (*ab* = −0.075) falls within a range that does not include zero (−0.037 to −0.012). Fig. 1 also indicates that the direct effect of DM on ABQ-RA is no longer statistically significant once the indirect effect of DM via SC is considered (*c*′ = −0.032, *p* = 0.421). In combination, DM and SC explain 18.3% (*F*_(2;153)_ = 17.107, *p* < 0.001) of the variance in the participants’ ABQ-RA scores.

The mediation analysis depicted in Fig. 2 indicates that DM is not indirectly associated with ABQ-E through SC. DM is related to an increase in SC (*a* = 0.455, *p* < 0.001). However, SC does not result in a significant change in ABQ-E (*b* = −0.004, *p* = 0.924). A 95% bias-corrected confidence interval based on 5 000 bootstrap samples indicates that the indirect effect (*ab* = −0.002) falls within a range that includes zero (−0.052 to 0.049). Moreover, the direct effect of DM on exhaustion remains statistically significant once the indirect effect of DM through SC is considered (*c*′ = −0.199, *p* < 0.001). Furthermore, DM and SC together account for 9.7% (*F*_(2;152)_ = 8.157, *p* < 0.001) of the variance in the sample’s ABQ-E scores.

Fig. 3 indicates that DM has an indirect effect on ABQ-D through SC. DM is associated with an increase in SC (*a* = 0.447, *p* < 0.001), and a reduction in ABQ-D is related to SC (*b* = −0.126, *p* = 0.001). A bias-corrected confidence interval based on 5 000 bootstrap samples indicates that the indirect effect (*ab* = −0.056) falls within a range that does not include zero (−0.099 to −0.022). It is further evident from Fig. 3 that the direct effect of DM on ABQ-D is no longer statistically significant once the indirect effect of DM via SC is considered (*c*′ = −0.085, *p* = 0.061). The combination of DM and SC explains 13.3% (*F*_(2;151)_ = 11.582, *p* < 0.001) of the variance in the participants’ ABQ-D scores.

## Discussion

The current study aimed to contribute to the growing literature on the mechanisms of mindfulness in sport psychology, specifically by exploring the extent to which SC mediates the interaction between DM and components of ABO among adolescent athletes.

DM demonstrated negative correlations with all three components of ABO. This finding is in line with the increasing evidence of the inverse relationship between DM and ABO in athlete populations. ^[4, 10–11]^ However, the current findings expand on existing research within the South African context by establishing that all three components of ABO demonstrate significant negative relationships to DM. This strengthens the case for focussing on DM as an important psychological construct in understanding ABO among adolescent athletes.

SC was positively associated with DM. This is in keeping with the prevailing opinion in the mindfulness and clinical literature that DM and SC seem to be conceptually and functionally related processes.^[7–8]^ Findings from the current study suggest that the relationship between SC and ABO might be somewhat more complex. SC was negatively associated with a reduced sense of athletic accomplishment and the sport devaluation components of ABO. However, no significant correlation was found between SC and the physical and emotional exhaustion (exhaustion) component of ABO. To date, only one other study appears to have directly explored the relationship between SC and ABO in an athlete population.^[10]^ Here significant relationships were found between SC and all components of ABO. It is important to note that the aforementioned study was conducted among athletes in a different cultural context and developmental stage than that of the participants in the current study. In addition, athletes in the two studies differed with respect to the sporting codes represented and level of competition. Consequently, findings with respect to the interaction between SC and exhaustion might be more reflective of the training load associated with a specific sport and/or level of competition. Further research is thus required before general conclusions can be drawn with respect to the exact relationship between SC and exhaustion.

In the current sample, SC was found to partially mediate the interaction between DM and a reduced sense of athletic accomplishment, as well as the interaction between DM and sport devaluation. Consequently, an increase in DM was related to greater SC, which was in turn associated with less of a sense of reduced athletic accomplishment and lower levels of sport devaluation among the participants. However, SC did not mediate the interaction between DM and exhaustion. Similarly, SC accounted for more of the variance in both the sense of reduced athletic accomplishment and sport devaluation when compared to DM, while DM accounted for more of the variance in exhaustion than did SC. Therefore, SC may be more salient to self-critical and evaluative cognitive processes involved in ABO, while being less relevant to somatic and intuitive components of ABO.

A sense of reduced athletic accomplishment is, at least partially, based on the subjective evaluation of the athlete’s current performance compared to their performance goals and/or the performance of other athletes.^[1,3]^ In a similar way, sport devaluation necessitates some form of cost-benefit analysis with regard to other aspects of the athlete’s life.^[1–3]^ It stands to reason that self-acceptance and self-kindness, as manifested in SC, would potentially undermine self-criticism and thus contribute to a lesser sense of reduced athletic accomplishment.^[7–9]^ Similarly, the value clarification, self-acceptance and sense of connection to others encompassed in SC would be expected to reduce the risk that adolescent athletes would devalue the role of sport in their lives.^[3, 7–9]^ Experiential acceptance, psychological flexibility, clarity regarding values, non-attachment and a reduced tendency to ruminate have been proposed as mechanisms via which mindfulness might facilitate athletic performance and athlete well-being.^[6]^ The similarity between these processes and those attributed to SC might provide insight into the manner in which SC mediated the impact of DM on a reduced sense of athletic accomplishment and sport devaluation in this study. While further research is required, it could be hypothesised that specific qualities or characteristics of mindfulness promote self-kindness and/or undermine self-criticism which, in turn, partially negates a sense of reduced athletic accomplishment and sport devaluation among adolescents.

The experience of physical and mental exhaustion within the context of ABO is conceivably less dependent upon evaluative cognitive processes or self-criticism. Mindfulness mechanisms, such as bare attention, as well as clarity with respect to physical sensations and internal experiences, are arguably more relevant to the experience of exhaustion.^[6]^ The finding that DM was associated with exhaustion, while SC was not associated with this component of ABO nor mediated its relationship to DM is thus not surprising. It could be speculated that SC does not have the same impact on exhaustion as it does on the other two aspects of ABO. However, adolescents competing in ball sports have been found to be at comparatively lower risk for ABO in relation to those involved in endurance, aesthetic, highly technical or weight-dependent sports.^[2]^ Moreover, few participants in this study compete at a particularly high level and most report low to moderate training loads. Consequently, findings related to exhaustion and SC or DM in this study should be interpreted circumspectly.

### Limitations

The study made use of a small sample of adolescents participating in one sport. Consequently, the reported findings cannot be generalised beyond adolescent athletes. Nor should they be extrapolated to other sporting contexts, particularly team sports or sporting codes that emphasise endurance, aesthetic performance, or require high levels of technical ability. There is a need for replication of this research in more varied athlete populations. Future research should explore the interrelationships between ABO, DM and SC across various sporting codes and contexts, as well as in a number of developmental stages and at different levels of competition.

To date, most of the research on SC within the sporting context has been conducted among female athletes. Furthermore, a recent study on ABO found differences between males and females with respect to SC and the mediation of the DM/ABO relationship by SC.^[10]^ The current study yielded no gender differences. However, given the small number of female participants, these findings should not be viewed as representative. Additional research is required to establish whether consistent gender differences are apparent with respect to ABO, DM and SC, as well as exploring the specific nature and possible effects of such differences.

The regression models tested in this study indicate that DM and SC explain only a small to moderate proportion of the variance in ABO among adolescent athletes. Moreover, where SC mediates the interaction between DM and ABO, this effect is only partial. DM and SC are thus not the only predictors of ABO, and SC is not the sole mediator of the effect of DM on ABO. More work is required to identify other predictors, as well as additional mediators and moderators with respect to the DM, SC and ABO relationship.

The current study employed a cross-sectional correlation design. Consequently, conclusions can only be drawn with respect to temporal and correlational relationships of the study variables to one another. Longitudinal studies are required to better understand the effect of incremental and developmental influences on the interaction between DM, SC and ABO. Experimental studies would be beneficial in two respects. Firstly, they would be better suited to exploring issues of causality with respect to the effects of DM and SC on ABO. Secondly, controlled experiments would allow for a more fine-grained examination of the mechanisms by which DM might influence athletic performance and athlete well-being.

## Conclusion

DM is significantly related to lower levels of ABO among adolescent athletes. However, SC appears to only be associated with a reduction in two components of ABO. SC partially mediates the interaction between DM and a reduced sense of athletic accomplishment, as well as the interaction between DM and sport devaluation. SC does not impact the relationship between DM and exhaustion. Mindfulness practices continue to hold promise in preventing and reducing ABO. However, based on the current study, the potential for tailored mindfulness interventions targeting specific components of ABO via SC should be explored further.

## Figures and Tables

**Table 1:** Correlations, reliability coefficients and descriptive statistics for the study variables (n=158)

Variables	CAMM	SCS-SF	ABQ-RA	ABQ-E	ABQ-D
CAMM	-	0.369**	−0.220**	−0.317**	−0.266**
SCS-SF		-	−0.431**	−0.127	−0.339**
ABQ-RA			-	0.172*	0.481**
ABQ-E				-	0.505**
*α*	0.750	0.794	0.679	0.872	0.739
*M*	21.45	39.39	11.96	9.72	8.57
*SD*	6.31	7.58	3.23	4.02	3.44

** indicates p < 0.01;

* indicates p < 0.05.

M, Mean; SD, Standard Deviation; CAMM, Child and Adolescent Mindfulness Measure; SCS-SF, Self-Compassion Scale – Short Form; ABQ-RA, Athlete Burnout Questionnaire – Reduced Sense of Athletic Accomplishment; ABQ-E, Athlete Burnout Questionnaire – Physical and Emotional Exhaustion; ABQ-D, Athlete Burnout Questionnaire – Sport Devaluation.

## References

[1] Raedeke TD Smith AL Development and preliminary validation of an athlete burnout measure J Sport Exerc Psychol 2001 23 4 281 306 10.1123/jsep.23.4.281 28682196

[2] Granz HL Schnell A Mayer J Risk profiles for athlete burnout in adolescent athletes: a classification analysis Psychol Sport Exerc 2019 41 1 130 141 10.1016/j.psychsport.2018.11.005

[3] Isoard-Gautheur S Guillet-Descas E Gustafasson H Athlete burnout and the risk of dropout among young elite handball players Sport Psychol 2016 30 2 123 130 10.1123/tsp.2014-0140

[4] Li C Zhu Y Zhang M Mindfulness and athlete burnout: a systematic review and meta-analysis Int J Environ Res Public Health 2019 16 3 449 10.3390/ijerph16030449 30717450 PMC6388258

[5] Rau HK Williams PG Dispositional mindfulness: a critical review of construct validation research Pers Individ Differ 2016 93 1 32 43 10.1016/j.paid.2015.09.035

[6] Birrer D Röthlin P Morgan G Mindfulness to enhance athletic performance: theoretical considerations and possible impact mechanisms Mindfulness 2012 3 3 235 246 10.1007/s12671-012-0109-2

[7] Hollis-Walker LH Colosimo K Mindfulness, self-compassion, and happiness in non-meditators: a theoretical and empirical examination Pers Individ Differ 2011 50 2 222 227 10.1016/j.paid.2010.09.033

[8] Neff K Self-compassion: an alternative conceptualization of a healthy attitude toward oneself Self Identity 2003 2 2 85 101 10.1080/15298860390129863

[9] Killham ME Mosewich AD Mack DE Women athletes’ self-compassion, self-criticism and perceived sport performance Sport Exer Perf Psychol 2018 7 3 297 307 10.1037/spy0000127

[10] Amemiya R Sakairi Y The role of self-compassion in athlete mindfulness and burnout: examination of the effects of gender differences Pers Individ Differ 2020 166 110167 10.1016/j.paid.2020.110167

[11] Walker SP Mindfulness and burnout among competitive adolescent tennis players S Afr J Sports Med 2013 25 4 105 108 10.7196/SAJSM.498

[12] Greco LA Baer RA Smith GT Assessing mindfulness in children and adolescents: development and validation of the Child and Adolescent Mindfulness Measure (CAMM) Psychol Assess 2011 23 3 606 614 10.1037/a0022819 21480722

[13] Walker SP Mindfulness and mental toughness among provincial adolescent female hockey players S Afr J Sport Med 2016 28 2 46 50 10.17159-516x/2016/v28i2a1110

[14] Raes F Pommier E Neff KD Construction and factorial validation of a short form of the Self-Compassion Scale Clin Psychol Psychother 2011 18 3 250 255 10.1002/cpp.702 21584907

[15] Cunha M Xavier A Castilho P Understanding self-compassion in adolescents: validation study of the Self-Compassion Scale Pers Individ Differ 2016 93 1 56 62 10.1016/j.paid.2015.09.023

[16] Hayes AF Introduction to mediation, moderation, and conditional process analysis: a regression-based approach New York Guilford Press 2013

[17] Foster JJ Parker I Carrying out investigations in psychology: methods and statistics Leicester Willey Blackwell 1995

